# Weight Management in Patients with Type 1 Diabetes and Obesity

**DOI:** 10.1007/s11892-017-0918-8

**Published:** 2017-08-23

**Authors:** Adham Mottalib, Megan Kasetty, Jessica Y. Mar, Taha Elseaidy, Sahar Ashrafzadeh, Osama Hamdy

**Affiliations:** 1 Joslin Diabetes Center, Harvard Medical School, Boston, MA 02215 USA; 2 Tufts University School of Medicine, Boston, MA 02111 USA; 3 Tufts University, Medford, MA 02155 USA; 4 One Joslin Place, Boston, MA 02215 USA

**Keywords:** Type 1 diabetes, Obesity, Weight management, Nutrition therapy, Anti-obesity medications, Bariatric surgery

## Abstract

**Purpose of review:**

Patients with type 1 diabetes (T1D) are typically viewed as lean individuals. However, recent reports showed that their obesity rate surpassed that of the general population. Patients with T1D who show clinical signs of type 2 diabetes such as obesity and insulin resistance are considered to have “double diabetes.” This review explains the mechanisms of weight gain in patients with T1D and how to manage it.

**Recent findings:**

Weight management in T1D can be successfully achieved in real-world clinical practice.

**Summary:**

Nutrition therapy includes reducing energy intake and providing a structured nutrition plan that is lower in carbohydrates and glycemic index and higher in fiber and lean protein. The exercise plan should include combination stretching as well as aerobic and resistance exercises to maintain muscle mass. Dynamic adjustment of insulin doses is necessary during weight management. Addition of anti-obesity medications may be considered. If medical weight reduction is not achieved, bariatric surgery may also be considered.

## Introduction

In the past 20 years, the prevalence of obesity has tripled worldwide, to the extent that it is now being considered an epidemic [1]. Obesity, defined as a body mass index (BMI) of ≥30 kg/m^2^, affects approximately 35% of men and 40% of women in the USA [2••]. It has recently been reported that obesity in particular is rising at a greater rate than overweight [3].

Though patients with type 1 diabetes (T1D) have traditionally been thought to have lower BMI, current research has shown otherwise [4]. The trend of increasing obesity prevalence has increased at a faster rate in patients with T1D compared to the general population [5]. Currently, around 50% of patients with T1D are either overweight or obese. They also have higher waist and hip circumferences when compared to healthy controls [4]. In the Pittsburgh Epidemiology of Diabetes Complications (EDC) Study, which followed adult patients with T1D for an average of 18 years, prevalence of overweight increased from 29 to 42% and prevalence of obesity increased sevenfold from 3 to 23% [5]. Weight gain appeared to be unrelated to aging and instead related to clinical factors such as insulin therapy [5].

Comorbidities, often associated with excess body weight, reduce the benefits of good metabolic control [6]. Thus, controlling body weight in patients with T1D is necessary due to the well-known relationship between obesity and cardiovascular disease (CVD) [7••]. Metabolic abnormalities related to obesity, such as the pro-inflammatory state, are likely to modify CVD risk in this population [7••]. So far, complications related to CVD have been the leading cause of mortality in patients with T1D [8]. In this review, we aim to describe the different mechanisms by which patients with T1D gain excess body weight and how clinicians can help them manage it.

## Mechanisms of Weight Gain

### Insulin Therapy

Insulin is anabolic hormone that plays a role in inhibiting protein catabolism, stimulating lipogenesis, and slowing basal metabolism [4, 5, 9], resulting in increased fat accumulation [9, 10]. Inhibiting protein catabolism is another anabolic process, in which weight gain may also occur through an increase in lean body mass [11]. These effects are enhanced by exogenous insulin administration [4], since exogenous insulin imperfectly mimics endogenous secretion [9, 12]. While endogenous insulin has its first pass to the liver through the portal vein to suppress gluconeogenesis [9], exogenous insulin circulates systemically first and disproportionately affects muscle and adipose in comparison to the liver [9].

#### Intensity of Insulin Therapy

Intensity of insulin treatment influences weight gain as shown in the Diabetes Control and Complications Trial (DCCT), where patients on intensive insulin therapy gained an average of 4.6 kg over 5 years, which is significantly more than patients in the study’s conventional arm [13]. In that study, participants treated with intensive insulin therapy administered insulin either by multiple daily injections (MDI) or through continuous subcutaneous insulin infusion (CSII) by insulin pumps [13]. Participants on conventional therapy administered one to two daily injections of intermediate and rapid-acting insulin, usually with no daily adjustments [13]. Weight gain was observed in the intensive insulin therapy cohort as a whole, regardless of MDI or CSII administration [13]. Similarly, a meta-analysis comparing multiple outcomes in adults with T1D using either MDI or CSII found no difference in weight gain [14]. Despite weight gain, intensive insulin treatment is the standard of care because of its strong clinical benefits such as reduction of glycated hemoglobin (HbA1c) and reduction of long-term microvascular complications [13]. Part of weight gain in association with intensive insulin therapy in T1D has traditionally been seen as normalization of weight by correcting for glycosuria, diuresis, and catabolism [5]. It was also noted that moderate weight gain did not negatively affect cardiovascular risk profile when associated with improved glycemic control [15]. This furthers the point that weight gain in patients with T1D on intensive insulin therapy is complex and multifaceted, but thus far a healthy balance must be reached.

#### Type of Insulin Therapy

Limited research had been conducted on how different types insulin and their methods of administration affect body weight in patients with T1D [16••]. However, multiple trials have shown that patients with T1D who used insulin detemir as the basal component of intensive insulin therapy maintained weight neutrality or even had small weight reductions over 1 year in comparison to NPH insulin [9, 10]. This could be related to its near-physiologic mechanism by inducing greater suppression of hepatic glucose output [17]. One study also showed that insulin glargine initially caused less weight gain than NPH insulin, but the difference disappeared after 1 year [10]. Weight gain may also be modified by insulin concentration. Two clinical trials comparing insulin glargine U300 to insulin glargine U100 in patients with T1D reported less weight gain over 6 months with insulin glargine U300, yet insulin requirements increased [16••].

#### Growth Hormone and Insulin-like Growth Factor-1

The non-physiological mechanism of exogenous insulin administration negatively affects growth hormone (GH) and insulin-like growth factor (IGF)-1, which help in maintaining the delicate balance between catabolism and anabolism [9]. Normally, GH regulates IGF-1 and counteracts insulin action by promoting lipolysis and inhibiting insulin-induced suppression of hepatic gluconeogenesis [12]. Higher concentration of GH blocks insulin signaling, resulting in elevation of both blood glucose and insulin concentrations [12]. Since patients with T1D are insulin deficient, hepatic IGF-1 synthesis is impaired resulting in decreased feedback suppression of GH and increases in GH concentration [12].

### Double Diabetes

Double diabetes is a new term used to describe patients with T1D who also show clinical signs of type 2 diabetes (T2D) such as obesity and insulin resistance (IR) [12, 16••]. With the rising rates of overweight and obesity among patients with T1D, there are no longer clear divisions between the two major diabetes subtypes except at time of diagnosis, as the disease appears to behave as a continuum with the two components of its etiology, insulin deficiency, and IR [12]. Double diabetes tends to occur when the pro-inflammatory state associated with metabolic syndrome leads to reduced glycemic control, eventually requiring higher daily doses of insulin [18•]. Increasing insulin dosage due to IR can lead to further weight gain, thus exacerbating the weight problem [18•]. Patients with T1D who are overweight or obese are at a greater risk of developing double diabetes due to their significantly elevated levels of osteopontin (OPN) [19]. OPN is a sialoprotein associated with normal physiological processes as well as autoimmune disease and has been demonstrated to induce adipose tissue inflammation, increase pro-inflammatory cytokine release, and promote development of IR [20]. Fortunately, weight loss reduces circulating OPN concentrations [19]. Double diabetes is a cyclical mechanism of weight gain and IR that should be recognized and treated early.

### Physical Inactivity

Increased physical activity to enhance weight loss is widely accepted, but adults with T1D tend to partake in less physical activity than adults without diabetes [21]. The main barrier to physical activity reported is fear of severe hypoglycemia [21]. Though this is a clear psychological barrier, it is also a valid concern since hypoglycemia is the most common adverse event of physical activity in patients with T1D [22]. Hypoglycemia may occur during or up to 24 h after activity [22]. To prevent hypoglycemia, patients usually reduce their insulin dose before exercise, but this strategy can only be used when exercise is planned in advance [23]. An additional drawback is that patients try to keep their blood glucose higher before exercise in order to maintain proper glycemic profile during and after exercise [23]. They do that by increasing consumption of carbohydrates before and during exercise, which results in increased energy intake and consequent weight gain [23].

## Weight Management in Type 1 Diabetes

### Nutrition Therapy

The American Diabetes Association recommends weight loss for all overweight or obese individuals with diabetes or at risk for diabetes [24]. Many nutrition-based approaches for weight loss have been studied in individuals with or without diabetes, but very few studies were specific to patients with T1D. For patients with T2D, certain macronutrient compositions such as low-carbohydrate or low-fat calorie-restricted diets and different eating patterns including Mediterranean and vegetarian dietary plans were shown to be successful for up to 2 years [24]. In a 2-year study comparing low-carbohydrate, low-fat, and Mediterranean dietary plans in obese participants, mean weight loss was 2.9 kg in the low-fat group, 4.4 kg in the Mediterranean diet group, and 4.7 kg in the low-carbohydrate group [25]. Among the 36 participants with T2D in the study, the Mediterranean diet, which is rich in vegetables and healthy fats and low in red meat, was the most favorable for changes in fasting plasma glucose and insulin levels [25]. The low-carbohydrate diet resulted in the greatest HbA1c reduction of 0.9% over 2 years [25]. Plant-based vegetarian or vegan diets [26] and the Dietary Approaches to Stop Hypertension (DASH) diet [27] have also been shown to induce weight loss and modest improvements in diabetes management. The low-fat vegan diet, devoid of all animal products, was associated not only with sustained weight reduction but also with reductions in total cholesterol and LDL-cholesterol in comparison to a cohort following the American Diabetes Association guidelines [26]. In a similar study, participants on a vegan diet had a decrease in HbA1c, attributed to loss of visceral fat [28]. Less restrictive vegetarian diets also promoted weight loss and reduced HbA1c [29]. The DASH diet, emphasizing vegetables, fruit, low-fat dairy, nuts, seeds, and whole grains while limiting meat, poultry, eggs, and oils, has shown beneficial effects on body weight, total and LDL-cholesterol, and insulin sensitivity [30].

Although these dietary plans, with different macronutrient compositions, have been shown to induce significant weight loss, the American Diabetes Association has determined in its position statement that there is no ideal macronutrient composition for meal plans. Current recommendations state that patients with diabetes should work with nutritionists to develop individualized eating plans based on the patient’s metabolic status, life circumstances, and food preferences [24].

Regardless of macronutrient breakdown, total energy intake must be appropriate to the weight management goal [24]. However, there are distinctions to be made in the quality of macronutrients and how they affect CVD risk factors and glycemic parameters [31•]. For carbohydrate consumption, intake of dietary fiber has been inversely associated with all-cause mortality in diabetes, while high glycemic load and sugar intake were associated with increased mortality [32]. In patients with T1D, meals with the same carbohydrate content but different glycemic indices produced significant differences in postprandial blood glucose, with low GI meals producing a 20% lower glycemic response than high GI meals [33]. For protein consumption, diets containing leaner sources of protein such as chicken and soy result in more favorable lipid profiles than diets containing red meat [34]. For fat consumption, type and source of fat are more important than the percentage or total amount of fat [35]. Diets containing foods high in monounsaturated fatty acids, such as extra-virgin olive oil and nuts, decreased CVD risk [36] and should therefore replace saturated and trans fatty acids [35].

### Increased Physical Activity and Exercise

Although weight loss can be achieved with only restriction of energy intake, increasing physical activity and incorporating exercise training into a weight loss plan lead to greater loss of fat mass and preservation of lean muscle mass compared to energy restriction alone [37, 38]. Additionally, there are metabolic benefits to partaking in physical activity for weight loss [37]. In patients with T1D, physical activity has been shown to decrease cardiovascular risk and mortality [39], in addition to improving lipid profile and endothelial function [40]. In patients with T2D, physical activity improves insulin sensitivity [39, 41]. As explained earlier, IR is not unique to those with T2D, as patients with T1D tend to be more insulin resistant than their counterparts without diabetes [39]. Therefore, the benefits of exercise on insulin sensitivity are pertinent to this population, especially in those who are overweight or obese.

Highly variable data exists as to what type of physical activity is best suited for weight reduction. Resistance training alone is associated with fat loss but has minimal effect on overall weight loss [42]. Even when resistance therapy is combined with aerobic training, this seems to lead to a similar amount of weight loss as aerobic training alone [37, 42]. One study showed that aerobic exercise was shown to lower visceral adipose tissue to a greater extent than progressive resistance training when compared to control groups [43]. However, the major benefit of resistance exercise is to preserve lean muscle mass during weight loss [44]. This is especially important since patients with diabetes have progressive lean muscle loss as they age [45].

In terms of exercise intensity, some studies have shown that high intensity interval training (HIIT), consisting of repeated bursts of rigorous exercise immediately followed by low intensity recovery, can lead to significant reductions in abdominal fat [46–48]. However, other evidence showed that while this approach is time efficient, it is no more effective than continuous moderate aerobic exercise in promoting fat loss [49]. This supports the observation that rigorous and moderate intensity aerobic training results in similar amounts of weight loss when intensities of physical activity are matched in energy expenditure [42]. Patients can partake in the type of physical activity they find most suitable as long as their energy expenditure is in line with their weight loss goals. Risk of hypoglycemia during or after exercise can be minimized if blood glucose is closely monitored before, during, and after exercise, and individual adjustments in insulin or food intake are made [50]. Patients with T1D should be safely able to participate in aerobic or weight-based physical activities if appropriate pre-exercise measures are taken [51••].

### Medications

#### Insulin

Adjustment of insulin treatment to facilitate weight reduction has been suggested [52]. Long-acting insulin creates a pattern of 24-h hyperinsulinemia, which stimulates lipogenesis and inhibits lipolysis [52]. Long-acting insulin such as NPH and glargine induce weight gain in patients with T1D [53]. If long-acting insulin is indicated, insulin detemir, insulin degludec, and insulin glargine U300 are preferred as they cause less weight gain compared to NPH or insulin glargine U100 [54–57]. To minimize the hypoglycemic risk and the unnecessary consumption of extra-calories, it is better to administer short-acting insulin immediately after meals or within 20 min from the start of the meal [58]. This gives patients the ability to calculate the short-acting insulin dosage based on the food that they actually consumed and not on what they presumed to eat. In patients with T1D, insulin glulisine is preferred in such scenarios due to its faster onset of action [58].

#### Metformin

Metformin is a potent anti-hyperglycemic agent used to treat T2D; however, several studies used metformin alongside intensive insulin therapy to treat patients with T1D and obesity [59, 60]. In a recent randomized control trial, patients with T1D using metformin had significant improvements in body weight and lipid profile over 3 years [61••]. While there was an initial reduction in HbA1c over the first 3 months of using metformin, this improvement was not maintained for over the next 33 months [61••]. However, these patients had a significant reduction in insulin dose requirements which is explained by metformin’s action as an insulin sensitizer [61••]. So far, US Food and Drug Administration (FDA) has not approved metformin for use in patients with TID.

#### Glucagon-like Peptide-1 (GLP-1) Analogs

GLP-1 is an incretin hormone that is involved in both peripheral and central pathways mediating satiation [62]. GLP-1 analogs are currently used to treat T2D and obesity. They reduce appetite and slow gastric emptying and thus reduce body weight and body fat by lowering energy intake [63]. Their use in patients with T1D resulted in significant weight reductions in overweight and obese patients [64••]. However, improvement in glycemic control did not reach statistical significance in trials using active comparators [64••]. Liraglutide, a GLP-1 analog, in conjunction with insulin has been shown to improve glycemic control and induce weight loss in patients with T1D [65, 66]. It was also found to reduce insulin dose [67]. While it is not approved for patients with T1D, its higher doses (2.4 and 3 mg/day) can be used to treat obesity. In a crossover study, exenatide treatment reduced postprandial plasma glucose but did not change HbA1c in patients with T1D [66, 68]. Another study showed that adding once weekly exenatide to insulin therapy significantly improved HbA1c, body weight, BMI, and reduced insulin doses [69, 70]. Currently, exenatide is not FDA-approved for use in patients with T1D.

#### Amylin Analog

Pramlintide is an injectable, synthetic form of human amylin [71]. Amylin is a beta cell hormone co-secreted with insulin and is nearly absent in patients with T1D [72•]. Amylin regulates blood glucose by slowing gastric emptying, suppressing glucagon secretion, and suppressing appetite to decrease food intake [72•]. Injecting pramlintide before meals in patients with T1D improves HbA1c, decreases postprandial blood glucose level, reduces insulin need, and induces weight loss [72•].

#### Sodium-Glucose Transporter-2 (SGLT-2) Inhibitors

This new class of medications reduces blood glucose by inhibiting glucose reabsorption in the proximal convoluted tubules of the nephrons [73]. Excretion of glucose in urine reduces body weight in addition to reducing HbA1c [74]. Recent studies showed cardiovascular benefits of two medications from this class; empagliflozin and canagliflozin [75, 76]. Several studies were done in patients with T1D showing reduction in plasma glucose and body weight but with increased incidence of ketoacidosis [77, 78]. Currently, this drug class is only FDA-approved for use in patients with T2D. Dual SGLT-1 and SGLT-2, sotagliflozin, is being investigated for use in patients with T1D [79•].

#### Anti-obesity Medications

There are four new anti-obesity medications approved recently by the US FDA (lorcaserine, topirmate/phentermine, neltroxone/bupropion, liraglutide). All of them plus the older medications like Orlistat and Phentermine are effective for weight loss with variable efficacy and side event profiles [80]. No studies using these medications were specifically conducted in patients with T1D. However, these medications showed reduction in HbA1c and number or doses of diabetes medications in patients with T2D [81–83]. It is not clear if this effect is related to weight loss or it is specific to the mechanisms of action of these medications.

## Bariatric Surgery

There are several surgical options to reduce body weight that are constantly suggested for patients with T2D [84]. The most commonly used methods are laparoscopic adjustable gastric banding (LAGB), sleeve gastrectomy (SG), and Roux-en-Y gastric bypass (RYGB) [84–88]. All of these methods were shown to significantly reduce body weight with variable duration [89–91] and improve glycemic control and may induce partial or complete remission from T2D, especially when they are done early in the course of the disease [92]. Several case series have been reported in obese patients with T1D showing reductions in body weight and insulin doses as well as a modest reduction in HbA1c [93••].

### Roux-en-Y Gastric Bypass

This procedure reduces stomach size by creating a small 15–30-mL gastric pouch [94]. While the mechanisms by which it improves blood glucose are only partially understood, there is good understanding of how it enables weight loss [94]. This surgery alters different gut hormone responses including GLP-1 and ghrelin, a potent hunger hormone [94]. After gastric bypass operations, GLP-1 secretion in response is significantly increased and this is presumed to contribute to the observed improvement in glycemic control [95–98]. It has been suggested that this surgery does not have a similar benefit on glycemic control if residual beta cell function is absent [99, 100].

A study comparing different types of bariatric surgeries found that complications are more likely to occur with RYGB than with sleeve gastrectomy [101]. Another study suggests that many patients who initially have remission from T2D relapse at some point after these procedures, particularly with RYGB and biliopancreatic diversion [87]. There is also a recognized risk of postprandial hypoglycemia [102] and weight regain after this procedure [103].

#### Sleeve Gastrectomy

Sleeve gastrectomy restricts stomach size by removing stomach fundus that contains cells that secrete ghrelin hormone. This results in a significant reduction in food intake and suppression of appetite [102, 104]. Now, it is the most commonly prescribed bariatric surgery due to its efficacy and durability in treating obesity and associated comorbidities [105]. Sleeve gastrectomy is associated with similar rates of complications as gastric RYGB [106].

A study that compared the effects of bariatric surgery in patients with T2D and T1D diabetes found that surgery could benefit T1D patients in terms of weight loss and improved glycemic control [107]. It was noted that after 1 year, the decrease in median HbA1c in patients with T1D was much less than in those with T2D [107]. In contrast, a few studies suggest that improved glycemic control may not be a probable outcome of bariatric surgery [108, 109].

## Conclusion

Prevalence of obesity has increased at faster rate in patients with T1D than in the general population. While intensive insulin therapy, lack of physical activity, and development of double diabetes explain some of the mechanisms for weight gain in patients with T1D, little is studied about effective interventions for weight management in this population who were portrayed for long as being lean. Dietary intervention, increased physical activity and exercise, adjustment of insulin therapy, adding other diabetes medications that positively impact body weight, or adding anti-obesity medications are suggested. If medical weight management fails, bariatric surgeries are valid methods for weight management in patients with T1D.
